# Association of genome variations in the renin-angiotensin system with physical performance

**DOI:** 10.1186/1479-7364-6-24

**Published:** 2012-11-24

**Authors:** Argyro Sgourou, Vassilis Fotopoulos, Vassilis Kontos, George P Patrinos, Adamantia Papachatzopoulou

**Affiliations:** 1 School of Science and Technology, Hellenic Open University, Patras, 262 22, Greece; 2 Laboratory of General Biology, Faculty of Medicine, University of Patras, Patras, 265 04, Greece; 3 Department of Pharmacy, School of Health Sciences, University of Patras, Patras, 265 04, Greece

**Keywords:** Genetic variations, Renin-angiotensin system, Physical performance

## Abstract

**Background:**

The aim of this study was to determine the genotype distribution and allelic frequencies of *ACE* (I/D), *AGTR1* (A +1166 C), *BDKRB2* (+9/−9) and *LEP* (G–2548A) genomic variations in 175 Greek athletes who excelled at a national and/or international level and 169 healthy Greek adults to identify whether some particular combinations of these loci might serve as predictive markers for superior physical condition.

**Results:**

The D/D genotype of the *ACE* gene (*p* = 0.034) combined with the simultaneous existence of *BDKRB2* (+9/−9) (*p* = 0.001) or *LEP* (G/A) (*p* = 0.021) genotypes was the most prevalent among female athletes compared to female controls. A statistical trend was also observed in *BDKRB2* (+9/−9) and *LEP* (G–2548A) heterozygous genotypes among male and female Greek athletes, and in *ACE* (I/D) only in male athletes. Finally, both male and female athletes showed the highest rates in the *AGTR1* (A/A) genotype.

**Conclusions:**

Our results suggest that the co-existence of *ACE* (D/D), *BDKRB2* (+9/−9) or *LEP* (G/A) genotypes in female athletes might be correlated with a superior level of physical performance.

## Introduction

Genetic polymorphisms that act as potential mediators of the human health and physical performance are targets for many research groups attempting to unravel their role to the genetic predisposition for a superior performance and endurance. There are up to 170 gene variant sequences, 17 mitochondrial DNA markers and 25 additional nuclear genetic markers in the human genetic map which are related to physical performance phenotypes as well as to good physical fitness [1].

One of the most extensively studied genome variations, widely associated with the human performance over the last decade, was the insertion (I) or deletion (D) of 287-bp *Alu* repeats within intron 16 of the angiotensin-converting enzyme (*ACE*) gene [rs1799752] [2,3]. *ACE* plays a key role along the biochemical pathway of the renin-angiotensin system (RAS), which controls the homoeostasis of the human circulatory system. Renin is a low molecular weight enzyme that is released by juxtaglomerular cells of the kidney in response to blood pressure failure. Renin converts its substrate angiotensinogen to angiotensin I, which is almost immediately converted by *ACE* to angiotensin II (AT II). AT II is a potent vasoconstrictor substance that acts mainly via AT II type-1 receptors. Also, *ACE* hydrolyses bradykinin which is a vasodilator, thus reduces peripheral resistance and hence blood pressure [4]. Additionally, RAS acts through other tissues as a paracrine/autocrine system [5], and its local activity in the cardiac [6], adipose [7] and skeletal muscular tissues [8] has been reported. It has been currently verified that the local adipose RAS is capable of functioning independently of the plasma RAS and it is up-regulated in obesity [9,10], where the presence of AT II stimulates leptin gene expression and secretion from adipocytes [11], revealing a considerable cross-interaction between leptin expression and RAS components. In particular, the leptin G–2548A promoter polymorphism (*LEP* G–2548A) [rs7799039] has been strongly associated with the serum leptin levels in overweight individuals and obesity and an increased risk for obesity [12-14]. A study with obese Zucker rats treated with *ACE* inhibitors have shown decreased leptin release [11], which supports the cross-interaction between leptin and *ACE* gene products. Recent results have shown that alterations in adipocyte production of the RAS components may contribute to disorders of the metabolic syndrome, including obesity and obesity-related hypertension and diabetes [15,16].

The presence of other polymorphisms, like the *AGTR1* (A +1166 C) allele in 3^′^ UTR of the AT II type-1 receptor gene [rs12721276], results in increased expression of the receptor gene [17], and the 9-bp deletion in exon 1 of the *BDKRB2* (β2 receptor of bradykinin) gene [rs72348790] results in a higher transcriptional activity and, consequently, to a quicker receptor's response to bradykinin molecules [18,19]. Additionally, the co-existence of the latter polymorphism with the *ACE* D/D genotype responsible for elevated *ACE* enzyme activity might counterbalance this activity, preventing bradykinin's hydrolysation, by withdrawing it in a higher rate.

In this study, we have investigated the presence of known polymorphisms named *ACE* (I/D), *AGTR1* (A +1166 C) and *BDKRB2* (+9/−9) along the RAS biochemical pathway as well as the one in the promoter of the *LEP* gene (G–2548A). Leptin exhibits a cross-interaction with RAS components. The presence of all the above polymorphisms in specific combination showed to play a role not only in the blood pressure control, but also in other metabolic pathways that might affect fitness and physical performance in humans.

## Results

### Genotypic distribution

For all four polymorphisms studied, among male athletes versus male controls, the highest percentages of male athletes appeared as heterozygotes for *ACE* (I/D), *BDKRB2* (+9/−9) and *LEP* (G/A) genes and homozygotes (A/A) for *AGTR1* gene polymorphism. In total, there were no statistically significant differences in genotypes between male athletes versus male controls (Table 1). However, *ACE* (I/I) genotype was absent in the international male athlete group of 39 out of 102 male athletes.

**Table 1:** Genotype distributions and allele frequencies of the four polymorphisms in athletes and control groups

		**Male controls**	**Male athletes**	***p*value**	**Female controls**	**Female athletes**	***p*value**
		**(*n*= 88)**	**(*n*= 102)**		**(*n*= 83)**	**(*n*= 73)**	
Genotype distributions							
*ACE*	II	12 (13.64%)	7 (6.86%)	0.121	14 (16.87%)	13 (17.81%)	0.877
	ID	45 (51.14%)	60 (58.82%)	0.288	*43 (51.81%)*	*25 (34.25%)*	*0.027*
	DD	31 (35.23%)	35 (34.31%)	0.859	*26 (31.33%)*	*35 (47.95%)*	*0.034*
*AGTR1* (A +1166 C)	AA	53 (60.23%)	57 (55.88%)	0.545	45 (54.22%)	41 (56.16%)	0.807
	AC	32 (36.36%)	39 (38.24%)	0.79	35 (42.17%)	26 (35.62%)	0.403
	CC	3 (3.41%)	6 (5.88%)	0.424	3 (3.61%)	6 (8.22%)	0.218
*BDKRB2* (+9/−9)	(+9/+9)	30 (34.09%)	33 (32.35%)	0.757	24 (28.92%)	20 (27.40%)	0.833
	(+9/−9)	46 (52.27%)	58 (56.86%)	0.53	45 (54.22%)	45 (61.64%)	0.349
	(−9/−9)	12 (13.64%)	11 (10.78%)	0.528	14 (16.87%)	8 (10.96%)	0.29
*LEP* (G–2548A)	AA	16 (18.18%)	19 (18.63%)	0.937	12 (14.46%)	15 (20.55%)	0.297
	GA	46 (57.27%)	58 (56.86%)	0.526	46 (55.42%)	41 (56.16%)	0.926
	GG	26 (29.55%)	25 (24.51%)	0.435	25 (30.12%)	17 (23.29%)	0.363
Allele frequencies							
I allele (*ACE* gene)		57 (64.8%)	67 (65.7%)	0.895	*57 (68.7%)*	*38 (52.1%)*	*0.034*
D allele (*ACE* gene)		76 (86.4%)	95 (93.1%)	0.121	69 (83.1%)	60 (82.2%)	0.877
A allele (*AGRT1* gene)		85 (96.6%)	96 (94.1%)	0.424	80 (96.4%)	67 (91.8%)	0.218
C allele (*AGRT1* gene)		35 (39.8%)	45 (44.1%)	0.545	38 (45.8%)	32 (43.8%)	0.807
+9 allele (*BDKRB2* gene)		76 (86.4%)	91 (89.2%)	0.548	69 (83.1%)	65 (89%)	0.29
−9 allele (*BDKRB2* gene)		58 (65.9%)	69 (67.6%)	0.800	59 (71.1%)	53 (72.6%)	0.833
A allele (*LEP* gene)		62 (70.5%)	77 (75.5%)	0.435	58 (69.9%)	56 (76.7%)	0.337
G allele (*LEP* gene)		72 (81.8%)	83 (81.4%)	0.937	71 (85.5%)	58 (79.5%)	0.316

Values in italics have significant *p* values.

In both female athletes and female controls, their genotypic distribution is shown in Table 1. In female groups, significant differences were apparent, such as the higher score in female athletes (47.95%) versus female controls (31.33%) (*p* = 0.034) of the *ACE* (D/D) genotype, while the *ACE* (I/D) genotype exhibited a higher score in female controls (51.81%) versus the female athlete group (34.25%) (*p* = 0.027). All other genotypic distributions did not reach statistical significance (Table 1). Furthermore, in the female athlete group that were homozygous for the *ACE* (D/D) genotype, the *BDKRB2* (+9/−9) or *LEP* (G/A) genotypes were more prevalent (*p* = 0.001 and *p* = 0.021, respectively), compared to the female control group (Table 2). Also, a significant difference was revealed in female athletes in the distribution of the *BDKRB2* (+9/+9) genotype (27.40%) versus that of the *BDKRB2* (−9/−9) genotype (10.96%) (*p* = 0.042). This trend was also observed in male athletes but did not reach statistical significance. Finally, the distribution of the *LEP* (A/A) and *LEP* (G/G) genotypes was similar among female athletes (20.55% and 23.29%, respectively).

**Table 2:** **Cross-tabulation: female athletes/female controls and *ACE *(D/D)/other genotypes**

		**Female controls**	**Female athletes**	***p*value**
		***ACE*** (**D/D**)	***ACE*** (**D/D**)	
*BDKRB2* (+9/−9)	(+9/−9)	*11 (13.3%)*	*26 (35.62%)*	*0.001*
*LEP* (G–2548A)	GA	*15 (18.1%)*	*25 (34.2%)*	*0.021*

Values in italics have significant *p* values.

### Allele frequencies

Allele frequencies concerning both cohorts of male athletes versus male controls and female athletes versus female controls are shown in Table 1. The frequency of each allele resulted from the sum of the homo- and the heterozygotes carrying the counted allele in their genotypes. A statistically significant higher percentage was noticed for the *ACE* I allele in female controls (68.7%) versus female athletes (52.1%) (*p* = 0.034).

### Allelic combinations

**Figure 1:**
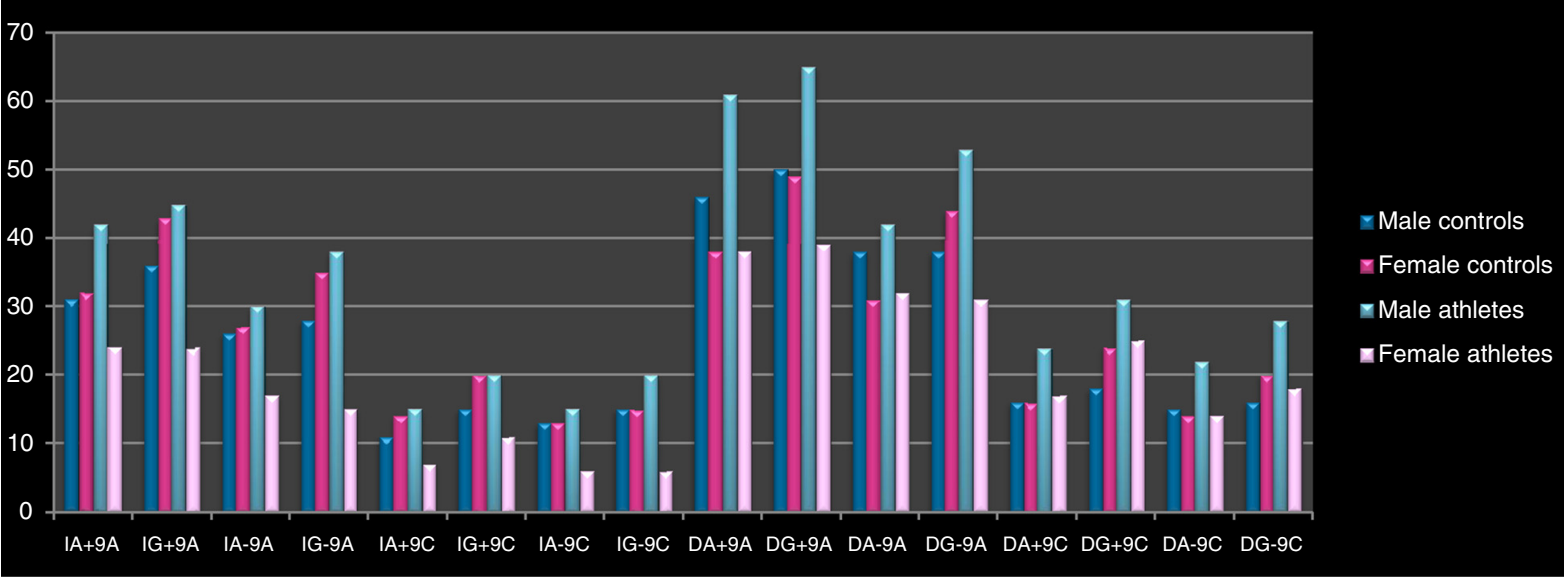
**Diagrammatic representation of the 16 allelic combinations among male/female controls and athletes.** The 16 allelic combinations resulted from all possible combinations of the eight different polymorphic alleles studied (*ACE* (I, D), *LEP* (G, A), *BDKRB2* (+9, −9) and *AGTR1* (A, C)).

The impact of the allele frequencies was further assessed by analysing the 16 possible allelic combinations coming from the four different studied polymorphisms (*ACE* (I/D), *LEP* (G/A), *BDKRB2* (+9/−9) and *AGTR1* (A/C); Table 3). Polymorphisms are referred as I or D, G or A, +9 or −9 and A or C. The percentages of each allelic combination quartet resulted from the use of the SPSS statistical program and the implementation of appropriate functions. Once more, significant differences were only observed in the female group (Figure 1). Specifically, the allelic combinations compared between female athletes and female controls revealed a significantly decreased frequencies of the IG+9A (32.9% versus 51.8%) and of the IG−9A (20.5% versus 42.2%) (*p* = 0.017 and 0.004, respectively) among female athletes.

**Table 3:** Allele combination frequencies of the four polymorphisms in athletes and control groups

***ACE *(I, D), *LEP *(G, A), *BDKRB2 *(+9, −9), *AGTR1 *(A, C)**	**Male controls**	**Male athletes**	***p *value**	**Female controls**	**Female athletes**	***p *value**
	**(*n*= 88)**	**(*n*= 102)**		**(*n*= 83)**	**(*n*= 73)**	
IA+9A	31 (35.22%)	42 (41.2%)	0.435	32 (38.6%)	24 (32.9%)	0.461
IG+9A	36 (40.9%)	45 (44.1%)	0.705	*43 (51.8%)*	*24 (32.9%)*	*0.017*
IA−9A	26 (29.54%)	30 (29.4%)	0.943	27 (32.5%)	17 (23.3%)	0.201
IG−9A	28 (31.8%)	38 (37.3%)	0.466	*35 (42.2%)*	*15 (20.5%)*	*0.004*
IA+9C	11 (12.5%)	15 (14.7%)	0.682	14 (16.9%)	7 (9.6%)	0.184
IG+9C	15 (17%)	20 (19.6%)	0.676	20 (24.1%)	11 (15.1%)	0.159
IA−9C	13 (14.77%)	15 (14.7%)	0.964	13 (15.7%)	6 (8.2%)	0.156
IG−9C	15 (17%)	20 (19.6%)	0.676	15 (18.1%)	6 (8.2%)	0.072
DA+9A	46 (52.27%)	61 (59.8%)	0.338	38 (45.8%)	38 (52.1%)	0.434
DG+9A	50 (56.8%)	65 (63.7%)	0.380	49 (59%)	39 (53.4%)	0.481
DA−9A	38 (43.2%)	42 (41.2%)	0.729	31 (37.3%)	32 (43.8%)	0.410
DG−9A	38 (43.2%)	53 (52%)	0.256	44 (53%)	31 (42.5%)	0.188
DA+9C	16 (18.18%)	24 (23.5%)	0.389	16 (19.3%)	17 (23.3%)	0.541
DG+9C	18 (20.45%)	31 (30.4%)	0.129	24 (28.9%)	25 (34.2%)	0.474
DA−9C	15 (17%)	22 (21.6%)	0.455	14 (16.9%)	14 (19.2%)	0.707
DG−9C	16 (18.18%)	28 (27.5%)	0.142	20 (24.1%)	18 (24.7%)	0.935

Values in italics have significant *p* values.

The same tests were applied throughout the groups of male athletes versus controls and showed no statistical significance (data not shown).

## Discussion

Physical performance seems to be controlled by many genetic factors that interact with the environment to affect complex interactions in human physical performance characteristics. All these render such investigations quite complex, and extended studies are needed to unravel possible interactions. To date, genetic studies, attempting to ascertain the role of genetic variants involved in the human superior physical performance, have focused on candidate genes mainly associated with cardiovascular functions, including *ACE* and proteins participating in skeletal muscle activity such as a-actinins [20,21]. Much of the mechanisms underlying the human athletic performance remain unexplored, despite 12 years of research on the most widely studied candidate polymorphic site of *ACE* I/D [3].

Both I and D alleles have been so far successfully associated in sports and with superior athletic performance in South African triathletes [22], British distance runners [23], swimmers [24] and sprinters [25]. The relationships between genotypes and performance, however, remain ambiguous. A recent study on 230 elite Jamaican and American sprinters found no association of either allele with sprint athlete status [26]. It has been suggested that at least part of the association of *ACE* with high athletic performance phenotypes is mediated through changes in kinin activity and is related to the existence of the *BDKRB2*(−9) allele as it provides a higher expression and abundance of the bradykinin receptor [27]. Besides the contribution of bradykinin to an impaired blood pressure, it also enhances insulin-stimulated tyrosine kinase activity of the insulin receptor, with subsequent GLUT-4 translocation in skeletal muscle tissue, thus giving a theoretical boosting effect during exercise [28]. Alternatively, the effects of the *ACE* (I/D) genotype may be mediated through changes in AT II activity, which acts via the *AGTR1* receptor. *AGTR1* (A +1166 C) gene polymorphism appears functional, with the C allele acting as an enhancer of the receptor activity; however, it does not seem to be associated with differences in high-level human performance [29].

In our study, we observed a strong statistical trend towards *ACE* (D/D) polymorphism among female athletes, which is in line with previous publications [24,25]. The highest rates of the corresponding group of male athletes appeared as heterozygotes (I/D) for the same polymorphism. Our results might not be in contrast with another study, which included 101 elite Greek track and field athletes [30]; this study suggested weak evidence that the presence of the *ACE* (D/D) genotype could influence sprint performance in Greek athletes. This might be due to both, i.e. the limited number of the participating athletes and the different kinds of sports they are considered elite.

Similar studies have shown that the *BDKRB2* (−9/−9) genotype was associated with the actual performance of 701 South African males who completed an Ironman Triathlon [19], but there were controversial results among Israeli athletes [31]. Likewise, a current study on the contribution of leptin gene promoter polymorphism *LEP* (G−2548A) in the human capacity for athletic performance indicated that the G allele provides an advantage to the reduction of body mass index as a response to physical training [32]. On the contrary, our data showed that female athletes of the *BDKRB2* (+9/+9) genotype were statistically higher than those of the *BDKRB2* (−9/−9) homozygotes, while a statistically significant increase was obtained for the co-existence of the *ACE* (D/D) genotype together with the heterozygosity of the *LEP* (G/A) or *BDKRB2* (+9/−9) genotypes (Table 2). Among female athletes, significant reduction of the I allele frequencies (Table 1) and of both IG+9A and IG−9A allelic frequency combinations (Table 3, Figure 1) were demonstrated, which are consistent with results coming from genotype distributions within the same group of athletes.

An interesting trend towards the heterozygous state of *ACE* (I/D), *LEP* (G/A) and *BDKRB2* (+9/−9) genotypes was also highlighted among male Greek athletes, which may require a larger sample size of athletes in order to be demonstrated. Finally, for the AT II type-1 receptor gene, both male and female athletes showed the highest rates in the *AGTR1* (A/A) genotype, but with no statistical significant results compared to the corresponding control groups.

New approaches should be identified to evaluate the impact of DNA polymorphisms in human fitness and high-level performance. One possible approach is conducting genome-wide association studies as the one performed by De Moor et al. [33], yet it is unclear whether their findings can be extrapolated to actual elite athletic status. Williams and Folland [34] in 2008 computed the ‘total genotype score’ (TGS), ranging from 0 to 100, resulting from the accumulated combination of 23 polymorphisms that are candidates to explain individual variations in endurance performance. Using a similar model, limited to seven well-studied polymorphisms associated with endurance capacity in Caucasians, Ruiz et al. [35] determined the actual TGS of the best Spanish male distance runners and road cyclists and suggested an overall more ‘favourable’ polygenic endurance profile in the athlete group than in Spanish normal individuals.

The limitation of our study is the small number of elite athletes, both as a whole and also in each sport. As such, this study can be considered a pilot investigation that can be expanded with the inclusion of further athletes. For the same reason, the abovementioned genomic markers have to be considered with caution and under no circumstances can be exploited to predict ones athletic performance. A meta-analysis study is currently under way to confirm or to overrule the predictive value of these biomarkers to assess athletic performance.

## Conclusions

The tendency for the heterozygous state in three: *ACE* (I/D), *BDKRB2* (+9/−9) and *LEP* (G/A), out of the four gene polymorphic sites studied was shown, but not proved by the statistical analysis in male athletes. Among female athletes, the co-existence of *ACE* (D/D) with *BDKRB2* (+9/−9) or *LEP* (G/A) genotypes and the reduction of I allele frequency and of both IG+9A and IG−9A allelic combinations were proved to be significant, compared to the female control group. Probably, a broader and more homogeneous sampling of athletes would demonstrate how strong the results highlighted in this study are and examine the effects of multiple genetic variants and allele combinations in superior physical performance.

## Methods

### Subjects and genotyping

One hundred and seventy-five Greek athletes and 169 Greek normal individuals who served as the control group were recruited. The athlete group consisted of 102 males and 73 females, whereas the control group consisted of 88 males and 83 females. Inclusion criteria for athletes were their participation and excellence at least once at international and/or national competitions, respectively. In particular, 39 out 102 men and 35 out 73 women had represented Greece at an international level in various sports: swimming (44 males:25 females), volleyball (16 males), handball (29 females) and athletics (long distance runners; 42 males:19 females).

DNA was collected with consent from 10 ml of peripheral blood or 10 ml of saline mouth rinse samples, and the DNA was isolated by QIAamp DNA Blood Kit (QIAGEN GmbH, Hilden, Germany). DNA amplification was performed with polymerase chain reaction, and subsequent restriction fragment length polymorphism analysis was carried out, according to the protocols described by Rigat et al. [36], Di Mauro et al. [37], Fischer et al. [38] and Mammes et al. [12] for *ACE* (I/D), *AGTR1* (A +1166 C), *BDKRB2* (+9/−9) and *LEP* (G−2548A) polymorphisms, respectively.

### Statistical analysis

Statistical analysis was performed with SPSS statistical software package (IBM SPSS Statistics version 19.0, Chicago, IL, USA). The statistical differences between groups in genotype distribution, allele frequencies and allelic combination frequencies are presented in Tables 1 and 3. The *p* values less than 0.05 were considered significant, and they were further assessed by Fisher's exact test.

We have statistically cross-tested genotypic distribution, allele frequencies and furthermore the frequencies of the 16 allelic combinations derived from the four genes examined (two different alleles per gene) between all groups. The tests were initially applied to the whole athlete group in comparison to the non-athlete group, and then each of the above groups were subdivided into cohorts of males and females where the same tests were applied again.

## Competing interests

The authors declare that they have no competing interests.

## Authors’ contributions

AS designed the study, collected the samples from (male/female) athletes and international teams, performed the experiments and drafted the manuscript. VF collected the data, carried out the statistical analysis and gave technical support and conceptual advice for the manuscript. VK participated in the design of the study, collected the control (male/female) samples and performed the initial experiments. GPP and AP developed the concept, designed the experiments and compiled the manuscript. AP also administered the experiments and supervised the statistical analysis. All authors read and approved the final manuscript.
